# Co-exposure of polyvinyl chloride microplastics with cadmium promotes nonalcoholic fatty liver disease in female ducks through oxidative stress and glycolipid accumulation

**DOI:** 10.1016/j.psj.2024.104152

**Published:** 2024-08-05

**Authors:** Yan Chen, Hengqi Jin, Waseem Ali, Tinglong Zhuang, Jian Sun, Tao Wang, Jie Song, Yonggang Ma, Yan Yuan, Jianchun Bian, Zongping Liu, Hui Zou

**Affiliations:** ⁎ College of Veterinary Medicine, Yangzhou University, Yangzhou, Jiangsu 225009, China; † Joint International Research Laboratory of Agriculture and Agri-Product Safety of the Ministry of Education of China, Yangzhou University, Yangzhou, Jiangsu 225009, China; ‡ Jiangsu Co-innovation Center for Prevention and Control of Important Animal Infectious Diseases and Zoonoses, Yangzhou, Jiangsu 225009, China

**Keywords:** PVC-MPs, Cd, Duck liver, NAFLD, Fibrosis

## Abstract

A recently discovered environmental contaminant, microplastics (**MP**) are capable of amassing within the body and pose a grave threat to the health of both humans and animals. It is widely acknowledged that the combination of cadmium (**Cd**), a hazardous heavy metal, and microplastics produces synergistic deleterious effects. Nevertheless, the mechanism by which co-exposure to polyvinyl chloride microplastics (**PVC-MP**) and Cd damages the liver of avian females is unknown. Globally prevalent and the subject of extensive research in mammals, nonalcoholic fatty liver disease (**NAFLD**) is a chronic liver condition. However, the mechanisms underlying injury to the avian digestive system caused by NAFLD remain unknown. Two months of co-exposure to Cd and PVC-MPs, pure water, solitary Cd exposure, single microplastics exposure, and pure water were administered to female Muscovy ducks in this study. The objective of this research was to examine whether the co-exposure of duck liver to PVC-MPs and Cd-induced oxidative stress resulted in NAFLD and subsequent apoptosis of hepatic cells. The study's findings showed that hepatocyte shape and functional activity were negatively impacted by PVC-MP and Cd buildup in liver tissues. Reduced liver organ coefficients, increased alanine aminotransferase (**ALT**) content, and ultrastructural damage to hepatocyte nuclei and mitochondria are indicators of this. These results point to a possible impairment in liver function. phosphoenolpyruvate carboxykinase 1 (**PCK1**) deficiency activates the protein kinase B/phosphatidylinositol 3-kinase (**PI3K/AKT**) pathway in the livers of female reproductive ducks that have been damaged by oxidative stress. This stimulation induces lipid deposition, fibrosis, and glycogen accumulation, which ultimately results in hepatocyte apoptosis. In summary, our research provides evidence that PVC-MPs cause oxidative harm to the liver, which subsequently results in fibrosis of liver tissue, hepatic glucolipid metabolism, and ultimately apoptosis.

## INTRODUCTION

The current state of plastic pollution is a matter of grave concern. Statistical evidence indicates that plastics are accumulating in the environment at a rate of 31.9 million tons per year and it is estimated that by 2050, more than 33 billion tons of plastics will have accumulated on the planet (Chelsea, 2018). Furthermore, given that plastics in the environment are inherently stable, it is challenging to degrade them through alterations to their physical and chemical properties, light, heat, temperature, and other environmental factors. Plastics can be degraded through continuous degradation into microplastics (**MP**, less than 5 mm in diameter) or lower-sized fragments (less than 100 nm) referred to as nanoplastics (**NP**). The pervasiveness of MPs in the environment renders human exposure to these particles an unavoidable inevitability (Prata et al., 2020). The presence of MPs was identified in a number of different products, including seafood, bottled water overpacks, beer, and household products (Banerjee and Shelver, 2021). The most common MPs found in the environment are Polystyrene (**PS**), polyethylene terephthalate (**PET**), high/low-density polyethylene (**PE**), polypropylene (**PP**), and polyvinyl chloride (**PVC**) (Colpaert et al., 2021; Jeyavani et al., 2022; Wagner et al., 2014). PVC-MP is one of the most common microplastics in the environment (Xu et al., 2020). PVC-MP is classified as a third most hazardous polymer because of its monomer carcinogenic properties and the incorporation of a large number of plasticizers in the production process (Lithner et al., 2011). Nevertheless, there is a paucity of research on PVC-MP.

There is accumulating evidence that MP may accumulate in various tissues and organs of terrestrial mammals, including the liver (Collard et al., 2017), kidneys (Exacoustos et al., 2023), ovaries (Li et al., 2022), testes (Zhao et al., 2023), among others. Previous studies have demonstrated that MPs can influence the hepatic metabolic and digestive system, with the most notable effects observed in oxidative damage, inflammation (Fu et al., 2023), lipid metabolism (Cheng et al., 2022), and hepatic fibrosis (Djouina et al., 2023). Furthermore, a recent study demonstrated the presence of MP in the tissues of patients with cirrhosis, whereas no MP were identified in the unaffected population (Thomas et al., 2022). Another study in workers revealed that PVC-MP possess a unique hepatic carcinogenic potential (Zarus et al., 2023). Nevertheless, there is a paucity of research investigating the toxicity mechanism of PVC-MP to the liver. This area of study requires further investigation. Cd is recognized as an environmental pollutant and the liver is one of its major target organs. The precise mechanism by which cadmium induces hepatotoxicity is not yet fully understood. However, it is thought that disruption of redox homeostasis and accumulation of reactive oxygen species (**ROS**) may play a role in this process, leading to apoptosis of hepatocytes (Bharati et al., 2020). The onset of liver disease initially manifests itself as excessive lipid deposits, known as NAFLD; although this is reversible, further deterioration often results in liver inflammation (**NASH**). A recent study suggests that Cd may play an important role in this process (Seogoo et al., 2022). Cd treatment was found to induce NASH, fibrosis and hepatocellular carcinoma in a study of preclinical models by Hyde et al. (Hyder et al., 2013). Therefore, there is a need to correlate Cd exposure with NAFLD studies.

Ducks represent a significant component of the poultry industry, and their aquatic nature necessitates the cultivation of environments with elevated water exposure. Consequently, ducks are more susceptible to environmental contamination than other poultry species (Liu et al., 2023a). Previous studies have demonstrated that MP possess the capacity to absorb a diverse array of environmental pollutants, which may potentially enhance their combined toxicity due to their considerable specific surface area and low surface polarity characteristics (Cao et al., 2021; Lu et al., 2018). Previous studies have demonstrated that liver injury induced by exposure of mice to polystyrene microplastics (**PS-MP**), either alone or in combination with Cd, is mediated by oxidative stress and apoptosis (Sheng et al., 2024). Additionally, it has been observed that co-exposure of Cd with MPs in mice promotes hepatic fibrosis through the ATP-P2×7 pathway, and that this process is synergistically affected by hepatic inflammation and fibrosis (Sun et al., 2023b). Cd is a toxic heavy metal. Due to its strong affinity for plastic materials, MP may be an effective carrier for Cd adsorption (Sun et al., 2023a). So, we expected that the relationship between MP and Cd would have an important effect on the liver toxicity. However, research on the combined MP and Cd to liver toxicity of avian species is limited, and more research is urgently needed.

The majority of current research on the co-exposure of MP and Cd is focused on aquatic animals. A previous study demonstrated that the concentration of Cd in the tissues of Callinectes sapidus that had been chronically exposed to MP (25 μg L^−1^) was found to be significantly elevated (Hernández-López, 2022). A further study on silver carp demonstrated that MPs have the capacity to enhance the bioavailability of Cd, with MP measuring 80 nm exhibiting a greater propensity for this phenomenon (Wang et al., 2022). The objective of this study was to investigate the impact of Cd exposure on the accumulation of PVC-MPs in female ducks. Additionally, the study aimed to explore the effects of co-exposure of PVC-MP and Cd on the hepatic digestive system, including the morphological and functional activity effects on the liver, oxidative stress injury, and its effects on NAFLD process. This process mainly included the detection of glycolipid metabolism, fibrosis, and apoptosis levels of hepatocytes. Ducks are frequently utilized as animal models for avian studies due to their diminutive size, pronounced sexual dimorphism, accelerated development, and adaptability. Additionally, duck meat and its byproducts, such as liver, represent a significant proportion of commercial production (Jian et al., 2021), underscoring the necessity for investigations into the toxicological mechanisms of liver injury in female breeding ducks by co-exposure of PVC-MP with Cd. The objective of this study is to elucidate the impact of PVC-MP and Cd exposure on the digestive system of birds, thereby enhancing our comprehension of the toxicological mechanisms underlying heavy metal contamination of avian livers. Additionally, the study will contribute to the understanding of the potential food safety risks and economic losses associated with heavy metal contamination in the poultry farming industry.

## MATERIALS AND METHODS

### Chemicals and Antibodies

Cadmium chloride (**CdCl2**) was acquired from Sigma-Aldrich, located in St. Louis, MO. The scanning electron microscope (Focus-Beam Technology, Beijing, China) was utilized to examine PVC-MPs provided by the Basesula Chromatography Technology Development Center (Tianjin, China). As shown in Table 1, these antibodies were used in the present study. Beyotime (Shanghai, China) supplied the hematoxylin and eosin stain kit as well as the period acid Schiff (**PAS**) stain reagent. Solarbio Beijing (Beijing, China) supplied the procurement of 3 stain kits: Masson's Trichrome, Modified Sirius Red, and Red Oil. The Jiancheng Bioengineering Institute supplied the following assay kits: total antioxidant capacity (**T-AOC**), glucose (**GLU**), malondialdehyde (**MDA**), Superoxide dismutase (**SOD**), catalase (**CAT**), reduced glutathione (**GSH**), ALT, pyruvate (**PYR**), and total antioxidant capacity (**T-AOC**). Additional reagents and compounds were procured locally and were of analytical grade.

**Table 1:** Antibodies used in the present study.

Antibody	Catalog number	Company
Bax	T40051	Abmart
Bcl-2	T40056	Abmart
Caspase-3	T40044	Abmart
Caspase-9	T40046	Abmart
AKT	60203-2-Ig	Proteintech
p-AKT	66444-1-1g	Proteintech
PI3K	60225-1-1g	Proteintech
α-SMA	80008-1-RR	Proteintech
TGF-β1	21898-1-AP	Proteintech
COL4A1	19674-0-AP	Proteintech
PCK1	sc-271029	Santa Cruz
GAPDH	66009-1-Ig	Proteintech
Goat antirabbit	7074	Cell signaling technology
Horse antimouse	7076	Cell signaling technology

### Animals and Chemotherapy

The measured concentrations of MPs in the middle and lower reaches of the Yangtze River in China were found to be around 1 mg/L, although certain regions displayed levels that surpassed 10 mg/L (Yin et al., 2023). As a result, 1 mg/L was chosen as the exposure concentration for PVC-MP. The experimental concentration of Cd utilized in this laboratory was established at 50 mg/kg, in accordance with literature reports and prior research (Ran et al., 2023). Women are more sensitive to Cd toxicity than men. So, female ducks were selected as experimental subject for *in vivo* experiments. Thirty-two healthy, 1-day-old female Muscovy ducks were applied in this study. One-day-old ducklings were procured from a nearby farm for the purposes of this research. Standard laboratory procedures were adhered to in order to maintain the temperature and humidity of the laboratory enclosures at 25°C ± 2°C and 72%, respectively. A total of 32 ducks, each group consisting of eight replicates, were allocated at random to 4 distinct treatment groups. Fresh water and a basal diet were provided to the control group (n = 8). A basal diet containing 50 mg/kg of Cd was administered to the Cd alone exposure group (n = 8). The MP alone exposure group (n = 8) was provided with a PVC-MP-containing basal diet. The basal diet of the Cd and PVC-MP co-exposure group (n = 8) comprised 50 mg/kg of Cd and 1 mg/L PVC-MPs in potable water. All ducks were confined in experimental cages that were subject to environmental control. During the course of 60 d, they were granted ad libitum access to food. The National Research Council (NRC, 1994) set specified requirements that were met by the nutrient content and amounts of the basal diet. For each group, feed intake and mortality were recorded daily, while duck body weight and feed intake were monitored weekly. The Animal Care and Use Committee of Yangzhou University granted approval for all procedures (Approval No. SYXK [Su] 2021-0027). Furthermore, every husbandry practice and euthanasia was conducted with utmost regard for animal welfare.

### Collection of Samples

Following a 60-d course of treatment, blood samples were collected from the mid-wing vein of ducks and centrifuged at 2,000 g for 15 min to obtain serum. All ducks were euthanized using a combination of xylazine (50 mg/kg) and ketamine (100 mg/kg) administered intramuscularly (**IM**). The liver was immediately removed and partially frozen in liquid nitrogen. The tissues were fixed in either 2.5% glutaraldehyde or 4% paraformaldehyde (**PFA**), or stored in a refrigerator at −80°C until further analysis.

### Bio-Chemical Analysis

A 10% tissue suspension is initially formulated by weighing a specific quantity of tissue in accordance with the results of the preliminary experiment. After 5 min at 1,000 g centrifugation, collect the supernatant for quantification. The cellular activities of ALT, MDA, SOD, CAT, GSH, T-GSH, GLU, PRY, LAC, and T-AOC were quantified in accordance with the manufacturer's instructions using commercial reagents sourced from the Nanjing Jiancheng Bioengineering Institute (Nanjing, China).

### Identification of PVC-MP in Liver Tissue by Immunofluorescence

To ascertain the content of PVC-MP in duck livers, duck livers were separated after 60 d of treatment with Cd and PVC-MP. They were then dehydrated in a 30% sucrose solution, frozen in liquid nitrogen by immersion in optical coherence tomography (**OCT**), and sliced into 5-μm-thick slices using a cryosectioner. Fluorescent PVC-MP in duck livers were observed by fluorescence microscopy (TCS SP8 STED, Leica).

### Determination of Cd, Cu, Zn and Mn in the Liver Tissue

At a temperature of 65°C, quantitative samples of duck liver tissue were desiccated in an oven. Then, using a mortar and pestle, the dry tissue samples were crushed into a powder and left overnight in a solution containing 1 mL of hydrogen peroxide and 5 mL of nitric acid. After a 2.5-h digestion process involving acid drive and microwave ablation, the dissolved solution was transferred to a constant volume vial and diluted to a volume of 20 mL using ultrapure water. It was subsequently extensively mixed via inversion. The atomic absorption spectrophotometer (PerkinElmer; Optima 3700DV) was employed to determine the concentrations of Cd, Cu, Zn, and Mn in liver tissue samples.

### Hematoxylin and Eosin Staining

All duck livers were surgically removed, trimmed, and immersed in a fixative solution containing 4% paraformaldehyde for a duration of 24 h, in accordance with established protocols. After discarding the fixative, the tissue was cleaned, allowed to dry naturally using a gradient of ethanol concentration, turned transparent using a xylene solution, and then impregnated with wax. Attaching sections of the tissue to slides was accomplished with a microtome. The methodology for hematoxylin and eosin staining was as described in the previous study (Sun et al., 2024). Hematoxylin/eosin was used to stain the cell nucleus and cytoplasm of tissue wax specimens that had been directly sectioned at a thickness of 5 µm. Every specimen was examined and documented utilizing a Leica light microscope DMI3000B (Leica, Wetzlar, Germany) outfitted with a digital camera.

### Periodic Acid Schiff Staining

After 5 min of treatment in periodic acid (5 g/L water), the tissue wax slides were rinsed with tepid water for an additional 5 min. Subsequently, the slides were stained with Coleman's Schi reagent for 5 min, followed by a 2-sec hematoxylin staining step. For microscopy analysis of duck liver glycogen content, the desiccated sections were rehydrated, rendered transparent, and subjected to imaging.

### BODIPY Staining

BODIPY, a lipophilic fluorescent probe designed to selectively bind to polar lipids, enables the labeling of neutral cellular lipid content, including lipid droplets in both living and fixed cells. Following the addition of PVC-MP and Cd, the cells underwent 2 PBS washes before being incubated for 20 min at 37°C with BODIPY dye. Prior to examination using a fluorescence microscope, the specimens underwent a triplicate washing process in phosphate buffered saline (**PBS**).

### Sirius Red Staining

The sections were incubated in a hematoxylin-iron solution for 5 min and in a Sirius red staining solution for 15 min. Following this, the sections were rinsed in running water, dried in ethanol, fixed in xylene, and sealed with neutral adhesive. The slices were observed in accordance with the aforementioned methodology (DMI3000B, Leica, Wetzlar, Germany).

### Masson's Trichrome Staining

The sections were stained with hematoxylin for a period of 5 to 10 min, after which they were washed with distilled water. The sections were then immersed in 2% aqueous glacial acetic acid and 1% aqueous phosphomolybdic acid for a period of 3 to 5 min each, after which they were stained with Lichon's red acidic magenta staining solution for a period of 5 to 10 min. The sections were then dried and incubated with aniline blue solution for a period of 5 min. The sections were then viewed under a microscope (DMI3000B, Leica, Wetzlar, Germany).

### Red Oil Staining

Following a PBS wash, liver tissue specimens were fixed for 8 to 10 min with 4% formaldehyde and then stained for 10 min with ORO staining (Solarbio) solution. The slides were subsequently rinsed for 15 min at 37°C in tepid distillate water. They were subsequently counterstained with hematoxylin for 2 min. to ascertain the lipid composition of liver tissue. Following rehydration and transformation to transparency, desiccated tissue sections were observed under a microscope.

### Transmission Electron Microscopy

As described in the preceding section (Vistro et al., 2019), tissue was prepared for TEM analysis. Following 2 PBS washes, the liver tissue was fixed in fresh 2.5% glutaraldehyde at room temperature for 2 h before being transferred to 4°C for 12 h. The specimens were observed through a transmission electron microscope after undergoing the following procedures: fixation in osmic acid, dehydration, entrapment, ultra-thin sectioning, negative staining, and observation.

### Scanning Electron Microscopy

First, the MPs were mixed with cadmium in a certain ratio (2:1) and homogenized overnight at 37°C on a shaker. After centrifugation at 12,000 g for 30 min on the second d, the MP were oven-dried at 65°C to powder, and the MP morphology was observed on the **SEM** (GeminiSEM 300, German).

### Immunohistochemistry

IHC was performed in accordance with earlier instructions (Shi et al., 2020). In preparation for histological examination, the tissue specimens were de-waxed in xylene for a duration of 2 min, followed by rehydration by submerging them in concentrated ethanol for ten min. PBS was used to wash tissue slides 3 times. In order to impede the activity of endogenous peroxidases, the sections were subjected to a 30-min treatment with 3% hydrogen peroxide (**H_2_O_2_**) in PBS at ambient temperature. Then, tissue slides were treated with a primary antibody and incubated in a moisture chamber at 4°C for overnight. Following washing, the sections were incubated with the secondary antibody for 1 h at room temperature. For fifteen min, the slices were exposed to DAB at room temperature (Boster Bio-Technology Co., LTD).

### TUNEL Staining

Terminal deoxynucleotidyl transferase-mediated dUTP-biotin nick end labeling (**TUNEL**) assay (Beyotime, China) was utilized in accordance with the manufacturer's guidelines to identify apoptosis in the injured liver sections embedded in formaldehyde-fixed paraffin-embedded sections.

### Western Blotting

To produce tissue homogenate from liver tissue samples, a specific volume of liver tissue should be weighed and lysate should be added in a 1:9 ratio. Subsequently, subject the mixture to ultrasound and centrifugation at 12,000 g for 15 min; the supernatant should be retained for further use. Bradford protein assay (PC0010, Solarbio) was used to determine the protein content. Proteins were transferred to polyvinylidene difluoride membranes (Millipore, MA) following separation by sodium dodecyl sulfate-polyacrylamide gel electrophoresis. After incubation with primary and secondary antibodies (PCK1, AKT, p-AKT, PI3K, Bax, Bcl-2, etc.), Image Laboratory software (Bio-Rad, Hercules, CA) was utilized to quantify the band densities.

### Statistical Analysis

A statistical analysis was performed on the data obtained from a minimum of 3 independent experiments, and the results were expressed as the mean with a standard deviation of SD. One-way analysis of variance (**ANOVA**) (Scheffe's SF test) was employed to examine the numerical data using GraphPad Prism 9 (Inc., La Jolla, CA), a new piece of software. A statistically significant difference was denoted by *P*-values below 0.05.

## RESULTS

### PVC-MP Accumulate in Duck Liver and Promote Cadmium Accumulation

**Figure 1:**
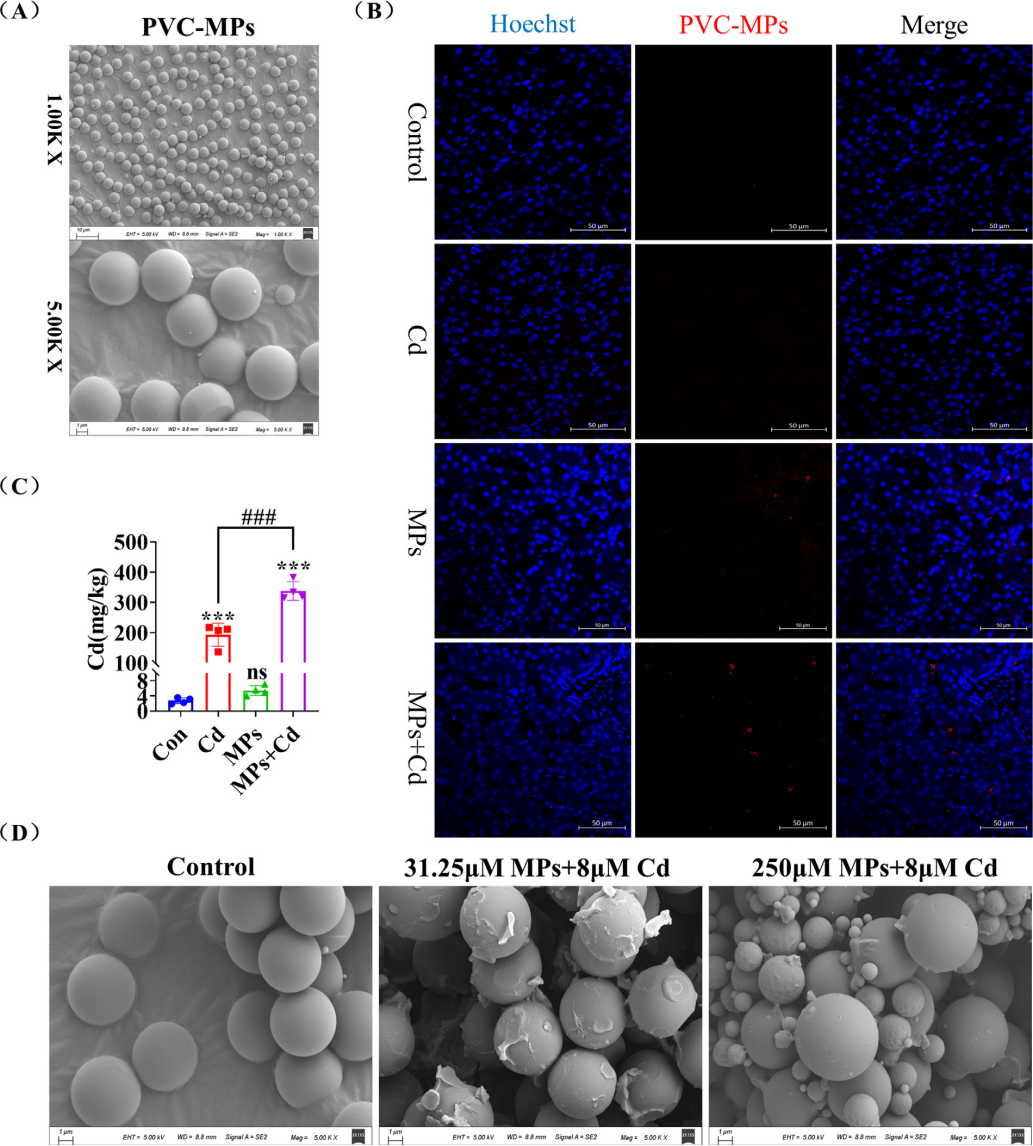
PVC-MPs accumulate in duck liver and promote cadmium accumulation. (A) Morphological characterization of PVC-MPs through SEM. (Scale bars = 10µm and 1µm). (B) Representative image of liver section from duck co-exposure to PVC-MPs and Cd for 60 d. Red fluorescence (**RFP**) indicates FPS-MPs. Blue fluorescence (Hoechst) represents the cell nucleus. (Scale bars = 50µm). (C) Determination of Cd in duck liver by graphite furnace atomic absorption spectrometry (**GFAAS**). (D) PVC-MPs were combined with Cd in a certain ratio, agitated at a minimum speed overnight, and the resulting morphological features were observed via SEM. (Scale bars = 10µm and 1µm). The values are expressed as the mean ± SD (n = 4). Note: “*” represents a statistically significant difference between the Cd group and the control group (*P < 0.05, **P < 0.01 or ***P < 0.001), “^#^” represents a statistically significant difference between the Cd group and Cd + MPs group (^#^*P* < 0.05, ^##^*P* < 0.01 or ^###^*P* < 0.001). The meaning of the “*” and “^#^” remains the same throughout the text.

First, the morphology of PVC-MP was observed using SEM. The results (Figure 1A) demonstrated that PVC-MP exhibited uniform particle size, smooth surface, and regular spherical particles. However, when PVC-MP were mixed with Cd at a certain ratio (Figure 1D), the surface of PVC-MP was observed to be rough and broken in the low concentration group, whereas the particle size of PVC-MP in the high concentration group was nonuniform and irregular spherical particles. Subsequently, we further evaluated the accumulation of PVC-MP in duck liver. As illustrated in Figure 1B, the red fluorescence of more positivity of PVC-MP was observed in the treated-liver frozen section of PVC-MP group and co-exposure of PVC-MP and Cd group, but not any clear positive evidence of PVC-MP concentration in the liver tissue of Cd group and control group. Furthermore, we investigated the impact of co-exposure of PVC-MP with Cd on the accumulation of Cd in duck liver tissues. Our findings (Figure 1C) demonstrated that this co-exposure resulted in a significantly higher accumulation of Cd in liver tissues compared to Cd exposure alone.

### Effect of Co-Exposure to PVC-MP and Cadmium on the Morphology and Functional Activity of Duck Liver

**Figure 2:**
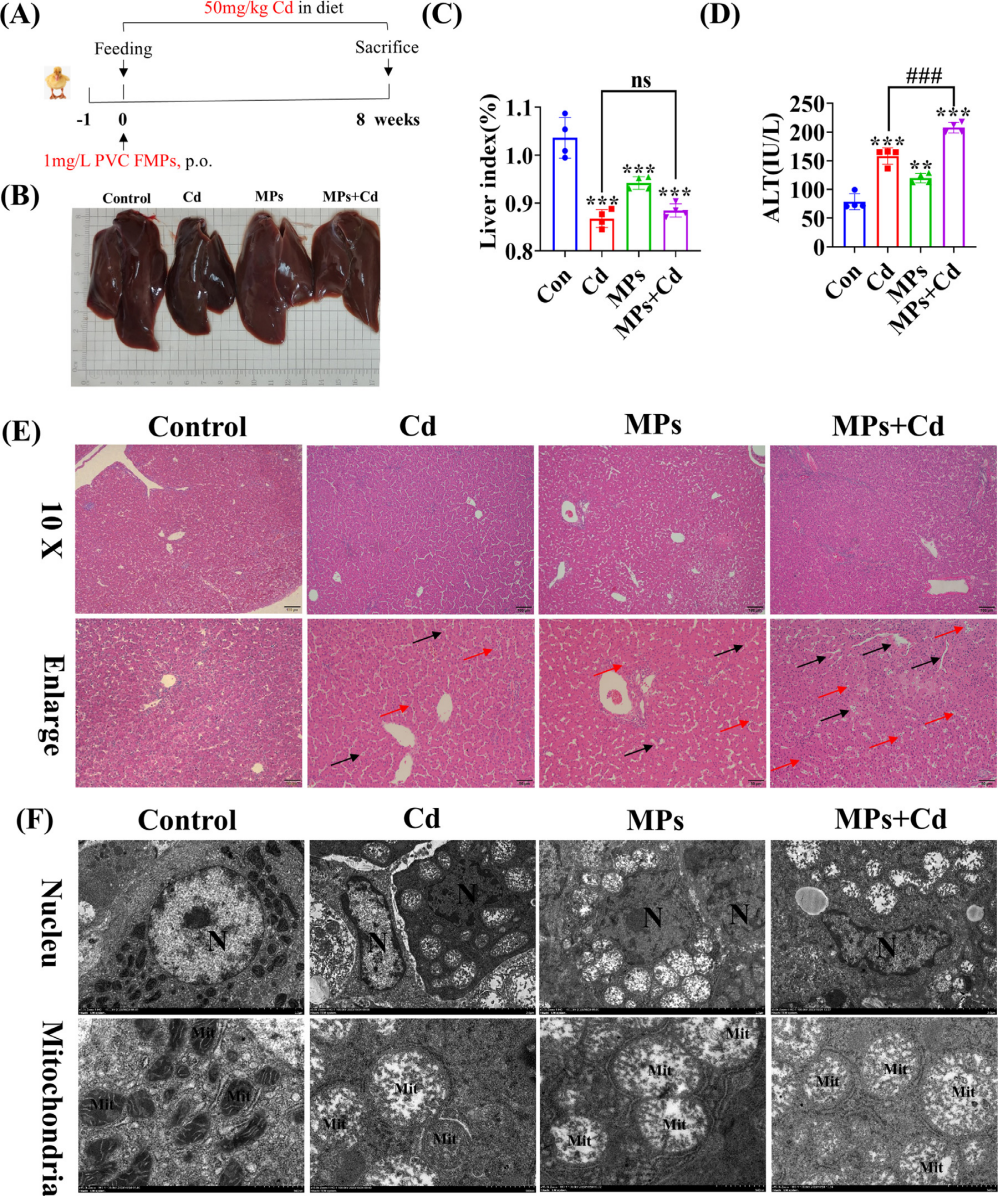
Effect of co-exposure to PVC-MPs and cadmium on the morphology and functional activity of duck liver. (A) Schematic diagram of animal experimentation process. Ducks were co-exposed to drinking PVC-MPs and dietary Cd for 60 d. (B) Morphological observation of the liver. (C) Effects of co-exposure to PVC-MPs and Cd for 60 d on liver coefficients of ducks. (D) Liver function was assessed according to serum levels of ALT. (E) HE staining of liver tissue of each group. Red arrows represent Kupffer cells (KCs). Black arrows indicate disorganized hepatic cords. (Scale bars = 100 µm and 50 µm). (F) Transmission electron micrograph was used to observe the nucleus and mitochondria in the liver tissue of each group. (Scale bars = 2µm and 500nm). The values are expressed as the mean ± SD (n = 4). Note: “*” represents a statistically significant difference between the Cd group and the control group (*P < 0.05, **P < 0.01 or ***P < 0.001), “^#^” represents a statistically significant difference between the Cd group and Cd + MPs group (^#^*P* < 0.05, ^##^*P* < 0.01 or ^###^*P* < 0.001). The meaning of the “*” and “^#^” remains the same throughout the text.

To verify the effects of co-exposure to PVC-MP and Cd on liver morphology and functional activity, we designed the following animal experiments: Ducks were co-exposed to drinking PVC-MP and dietary Cd for 60 d (Figure 2A). Morphological observations of the appearance revealed that the liver tissue of ducks was significantly smaller in the exposed group alone and in the co-exposure to PVC-MP and Cd group compared to the control group (Figure 2B). Furthermore, the organ coefficients yielded consistent results (Figure 2C). Additionally, serum levels of ALT, a marker of liver function were significantly elevated compared to the control group (Figure 2D). Compared to the cadmium-alone exposure group, HE staining showed that co-exposure to PVC-MPs and Cd group exhibited more severe hepatic medullary disorders and inflammatory cell infiltration (Figure 2E). Interestingly, significant lipid droplet vacuoles appeared in the co-exposed group, and we speculated that co-exposure to PVC-MPs and Cd might induce lipid accumulation in duck liver.

In addition, the ultrastructural components of hepatocytes in the duck were examined, utilizing transmission electron microscopy. The hepatocytes in the control group exhibited typical ultrastructural characteristics, including a regular nucleus morphology and fully developed mitochondria. On the other hand, the hepatocytes' nucleus and mitochondria displayed morphological alteration in the treated groups. In contrast, the co-exposure to PVC-MP and Cd group exhibited a more constricted nuclear membrane encircling the nucleus and an enlarged nuclear pore opening in comparison to the cadmium-alone exposure group. Conversely, the mitochondria found in the group exposed only to cadmium alone seemed to be extremely crowded, with a broken outer membrane. In the meantime, hepatocytes that had been co-exposed to PVC-MP and the Cd group displayed fractures, disintegrated mitochondrial cristae, and increased vacuolization inside their mitochondria (Figure 2F). In addition, a significant infiltration of lipid droplets was observed in the co-exposed group, which was consistent with the findings of HE staining. The findings of this study indicate that the concurrent exposure of duck liver to PVC-MP and Cd had an adverse impact on its development, morphology, and functional activity.

### Effect of Co-Exposure to PVC-MP and Cadmium on Oxidative Stress in Duck Liver

**Figure 3:**
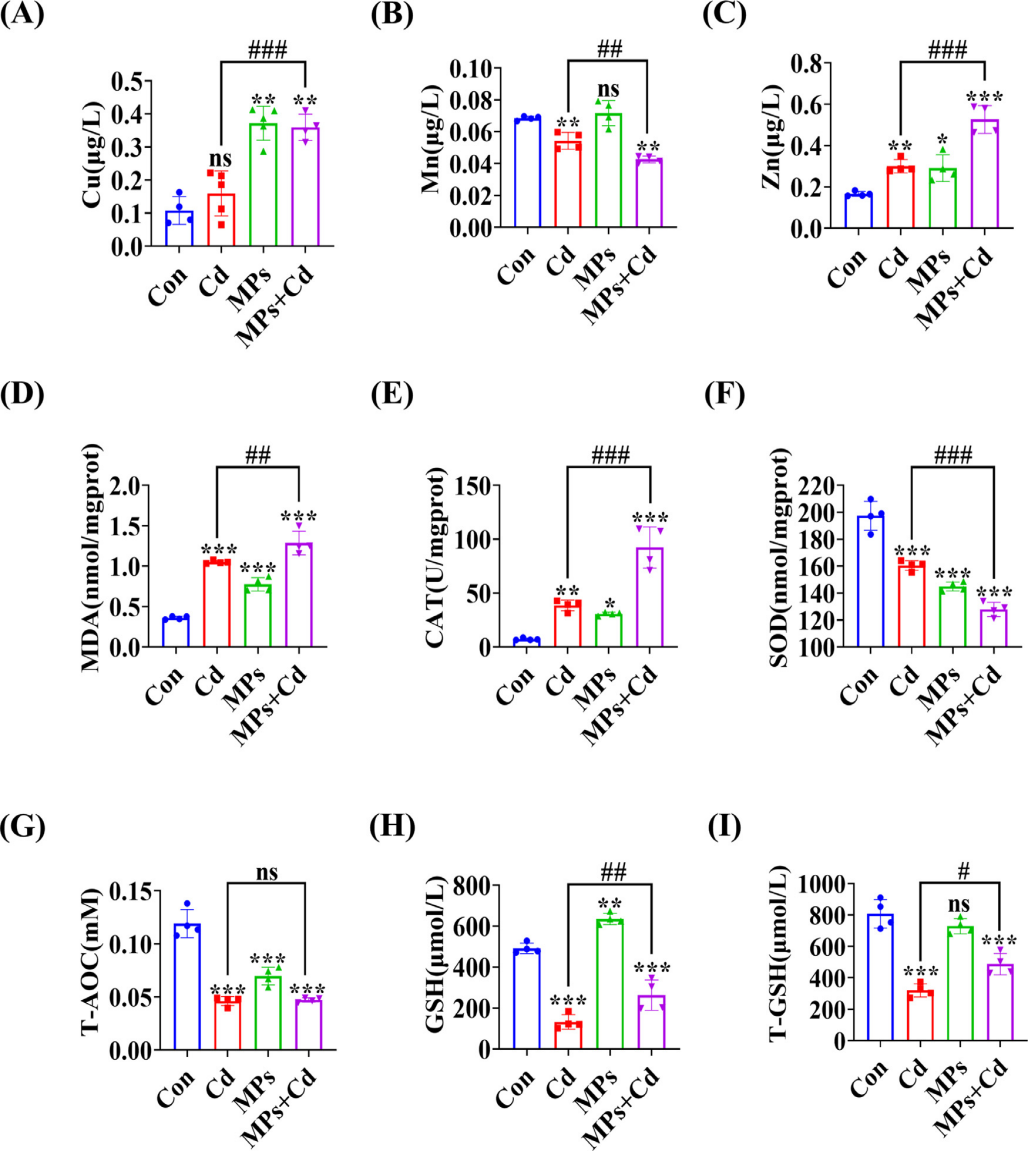
Co-exposure to PVC-MPs and cadmium promote oxidative stress in duck liver. Ducks were co-exposed to drinking PVC-MPs and dietary Cd for 60 d. (A-C) Cu, Mn, and Zn contents in dried liver tissue were determined through flame atomic absorption spectroscopy (**FAAS**). (D-I) Measurement of oxidative enzymes level of MDA, and anti-oxidative enzymes level of and CAT, SOD, T-AOC, GSH and T-GSH in the liver tissue of different groups. The values are expressed as the mean ± SD (n = 4). Note: “*” represents a statistically significant difference between the Cd group and the control group (*P < 0.05, **P < 0.01 or ***P < 0.001), “^#^” represents a statistically significant difference between the Cd group and Cd + MPs group (^#^*P* < 0.05, ^##^*P* < 0.01 or ^###^*P* < 0.001). The meaning of the “*” and “^#^” remains the same throughout the text.

It is imperative to acknowledge that oxidative damage, characterized by an overabundance of ROS, is regarded as the principal toxicological mechanism (Liu et al., 2022). For 60 d, ducks were supplemented with dietary Cd and consumed PVC-MP. The concentrations of Cu, Zn, and Mn in desiccated liver tissue were determined utilizing a flame atomic spectrophotometer. The findings demonstrated that, in comparison to the control and Cd groups, the co-exposure PVC-MP and Cd group considerably raised the levels of Cu and Zn and lowered the levels of Mn in the liver tissue (Figures 3A-C). The liver tissue exhibited oxidative and antioxidative enzyme activity subsequent to co-exposure to PVC-MP and Cd. The findings indicated that the co-exposure group to PVC-MP and Cd had significantly reduced levels of T-GSH, T-AOC, and SOD, in comparison to the control group. However, the group exposed to Cd and MDA exhibited significantly elevated levels of CAT and T-GSH (Figures 3D-I).

### Effect of Co-Exposure to PVC-MP and Cadmium on Lipid Accumulation in Duck Liver

**Figure 4:**
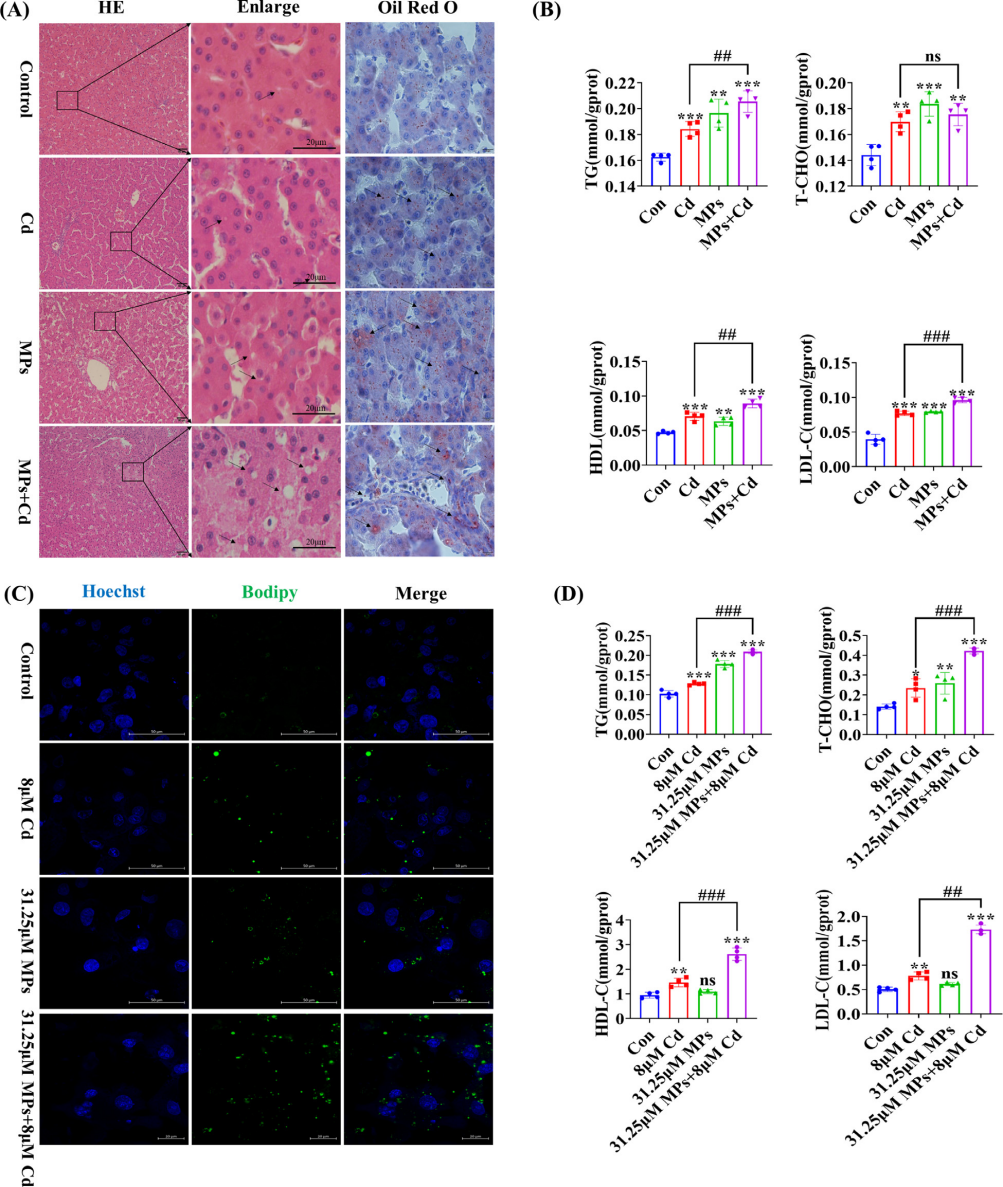
Co-exposure to PVC-MPs and cadmium promote lipid accumulation in duck liver. (A) Ducks were co-exposed to drinking PVC-MPs and dietary Cd for 60 d. HE and red oil staining were used to detect the level of lipid deposition in liver tissue of each group. Black arrow indicates lipid droplets. (Scale bars = 20µm and 10µm). (B) TG, T-CHO, HDL-C, and LDL-C levels in liver tissue of each group. (C) PDH was exposed to 31.25 μM PVC-MPS and 8 μM Cd alone or in combination for 12 h. BODIPY staining were used to detect the level of lipid deposition in PDH of each group. Green fluorescence (**GFP**) represents droplet. Blue fluorescence (Hoechst) represents the cell nucleus. (Scale bars = 50 µm and 100 µm). (D) TG, T-CHO, HDL-C, and LDL-C levels in PDH of each group. The values are expressed as the mean ± SD (n = 4). Note: “*” represents a statistically significant difference between the Cd group and the control group (*P < 0.05, **P < 0.01 or ***P < 0.001), “^#^” represents a statistically significant difference between the Cd group and Cd + MPs group (^#^*P* < 0.05, ^##^*P* < 0.01 or ^###^*P* < 0.001). The meaning of the “*” and “^#^” remains the same throughout the text.

To verify the effect of co-exposure to PVC-MPs and Cd on lipid accumulation in duck liver tissue, HE and Red Oil staining were performed. The results of the HE staining indicated a significant increase in the number of lipid droplet vacuoles in the liver of the co-exposure PVC-MP and Cd group compared to the Cd group. Red Oil staining yielded consistent results (Figure 4A). Additionally, the results of the lipid metabolism-related enzyme kit assay indicated that the TG levels in the liver were significantly higher in the co-exposure PVC-MP and Cd group compared to the Cd group. Furthermore, the levels of high-density lipoprotein (**HDL**) and low-density lipoprotein (**LDL**) were also affected (Figure 4B).

Additionally, primary duck hepatocytes (**PDH**) were treated with 31.25 μM PVC-MPS and 8 μM Cd for 12h. PDH yielded the same results. BODIPY staining revealed a significant increase in the number of lipid droplets in the hepatocytes of the co-exposure PVC-MP and Cd group compared to the Cd group (Figure 4C). The kit for lipid metabolism-related enzymes revealed a significant increase in intracellular TG and T-CHO content in the hepatocytes of the co-exposure PVC-MP and Cd group compared to the Cd group. Additionally, there was a significant increase in HDL and LDL content (Figure 4D).

### Co-Exposure of Duck Livers to PVC-MPs and Cadmium Regulates Glycogen Accumulation Through the PCK1-PI3K/AKT Pathway

**Figure 5:**
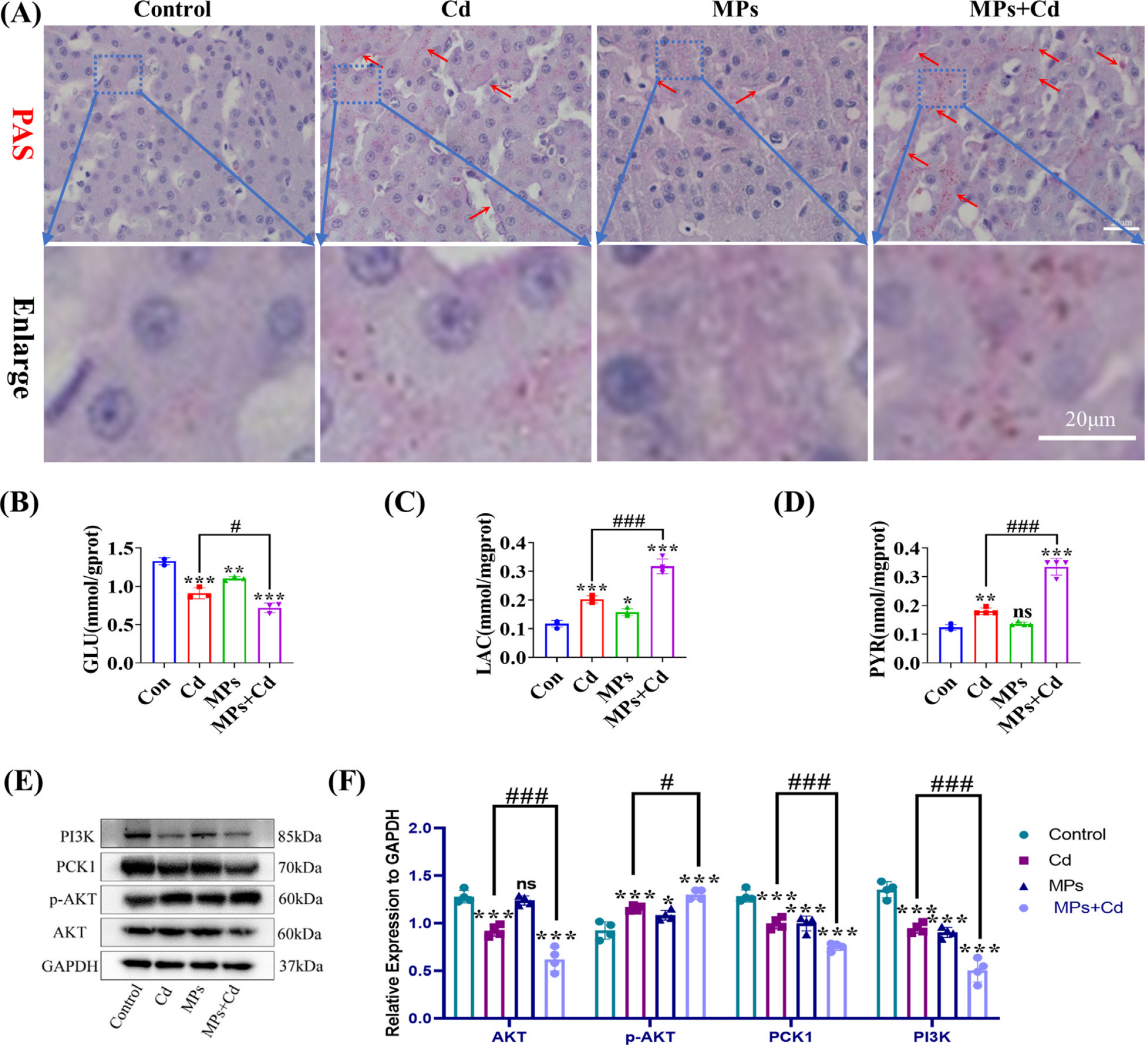
Co-exposure of duck livers to PVC-MPs and cadmium regulates glycogen accumulation through the PCK1-PI3K/AKT pathway. Ducks were co-exposed to drinking PVC-MPs and dietary Cd for 60 d. (A) PAS of liver tissue in each group. (B-D) GLU, LAC, and PYR levels in liver tissue of each group. (E-F) Levels of PCK1, PI3K, AKT, and p-AKT were determined using western blotting. (E) Representative western blot image. (F) Quantitative analysis of E. The values are expressed as the mean ± SD (n = 4). Note: “*” represents a statistically significant difference between the Cd group and the control group (*P < 0.05, **P < 0.01 or ***P < 0.001), “^#^” represents a statistically significant difference between the Cd group and Cd + MPs group (^#^*P* < 0.05, ^##^*P* < 0.01 or ^###^*P* < 0.001). The meaning of the “*” and “^#^” remains the same throughout the text.

To investigate the potential mechanisms by which PVC-MPs regulate duck liver, we collected liver from ducks that were fed 5 μm PVC-MP and Cd for 60 consecutive d. The results of PAS indicated that co-exposure of PVC-MP and Cd resulted in a significant increase in duck liver glycogen compared with the Cd group (Figure 5A). The results of the kit assay demonstrated that co-exposure of PVC-MP and Cd significantly elevated the levels of PYR and LAC and significantly decreased the levels of GLU in comparison to the Cd group (Figures 5B-D). This may be attributed to the inhibition of oxidative metabolism of glucose by co-exposure, which subsequently led to the glycolytic pathway. The results of the Western Blotting assay demonstrated a notable decline in PCK1, PI3K, and AKT protein expression levels and a pronounced elevation in p-AKT protein expression in the co-exposed group in comparison to the Cd group (Figures 4E-F). The above results indicate that co-exposure of duck livers to PVC-MP and cadmium regulates glycogen accumulation through the PCK1-PI3K/AKT pathway.

### Effect of Co-Exposure to PVC-MP and Cadmium on Liver Fibrosis

**Figure 6:**
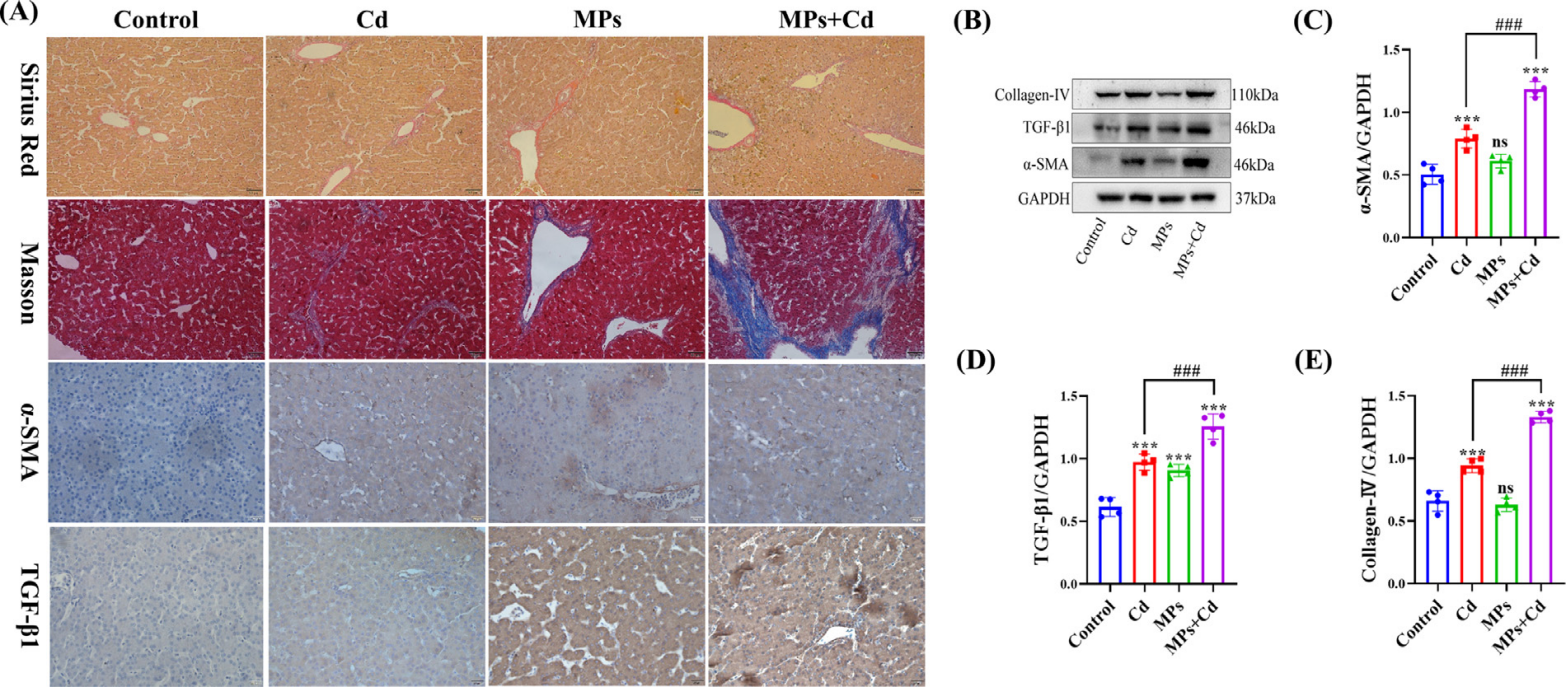
Co-exposure to PVC-MPs and cadmium promote liver fibrosis. Ducks were co-exposed to drinking PVC-MPs and dietary Cd for 60 d. (A) Sirius Red staining and Masson staining were used to detect collagen fibers in liver tissue of each group. Immunohistochemistry was used to detect expression level of α-SMA and TGF-β1 in liver tissue of each group. (Scale bars = 50 µm and 20 µm). (B-E) Levels of α-SMA, TGF-β1, and Collagen-Ⅳ were determined using western blotting. (B) Representative western blot image. (C-E) Quantitative analysis of B. The values are expressed as the mean ± SD (n = 4). Note: “*” represents a statistically significant difference between the Cd group and the control group (*P < 0.05, **P < 0.01 or ***P < 0.001), “^#^” represents a statistically significant difference between the Cd group and Cd + MPs group (^#^*P* < 0.05, ^##^*P* < 0.01 or ^###^*P* < 0.001). The meaning of the “*” and “^#^” remains the same throughout the text.

In order to ascertain the impact of co-exposure to PVC-MPs and Cd on collagen fibers in liver tissue, immunohistochemistry, Sirius red staining, and Masson staining were employed. The results of Sirius Red and Masson staining indicated that the Cd group exhibited a greater abundance of positive collagen fibers than the control group. In contrast, the collagen fibers in the co-exposure to PVC-MP and Cd group were stained more positively than in the Cd group alone. Furthermore, the immunohistochemistry assay findings indicated that the administration of Cd treatment resulted in a greater expression of TGF-β1 andα-SMA in the liver tissue when compared to the control group. On the other hand, compared to the Cd group, the co-exposure to PVC-MP and the α-SMA immunosignaling was much higher (Figure 6A).

Additionally, Western blotting was conducted on liver tissue from each group in order to validate the protein expression associated with fibrosis. The findings of the study revealed that the protein expression levels of COL4A1, TGF-β1, andα-SMA were significantly elevated in the Cd group as compared to the control group. In contrast to the Cd group, the co-exposure to PVC-MPs and the group experienced more severe alterations in fibrosis-related protein markers (Figures 6B-E). These results indicate that hepatic fibers are caused by co-exposure to PVC-MP and Cd.

### Effect of Co-Exposure to PVC-MP and Cadmium on Apoptosis in Duck Liver

**Figure 7:**
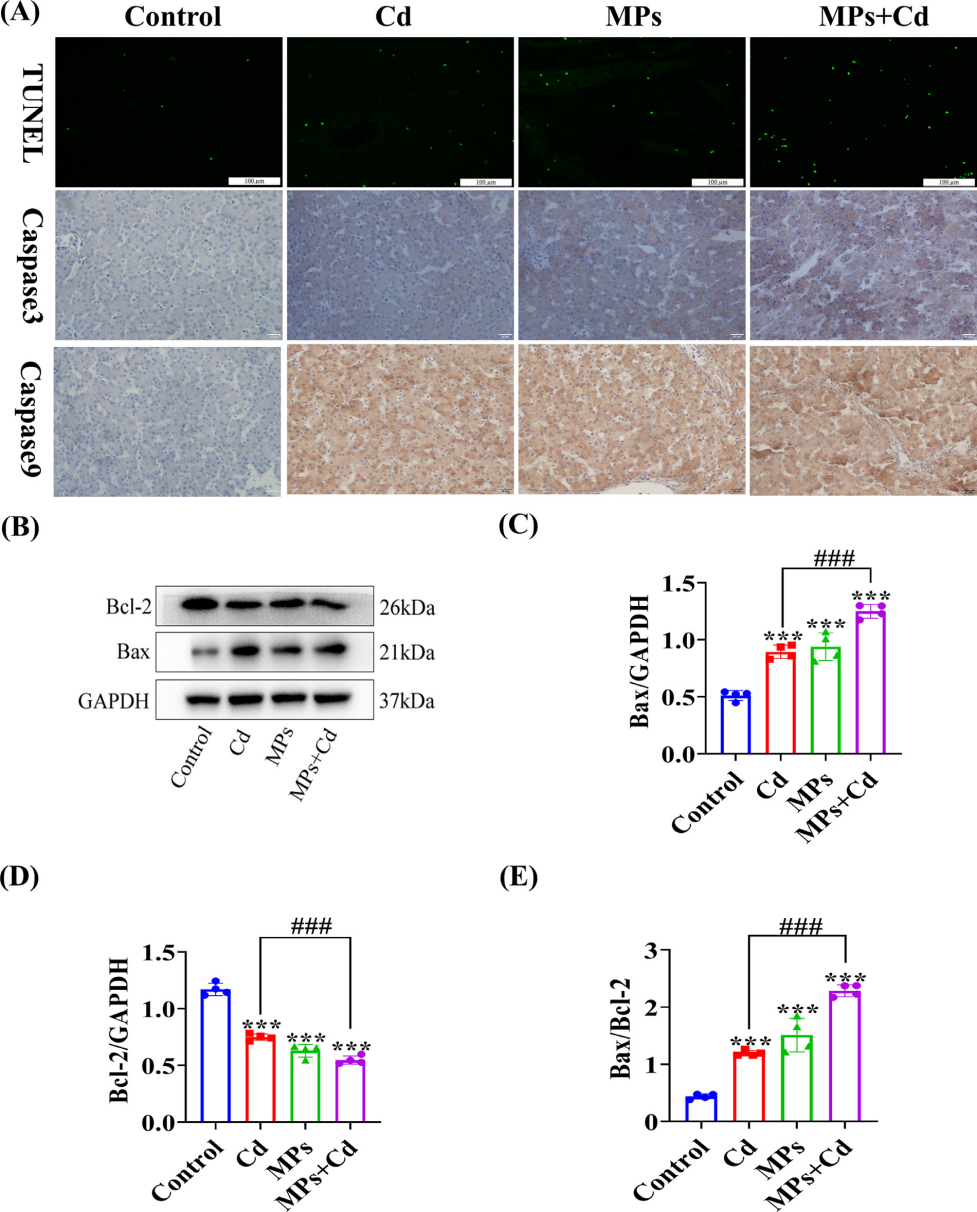
Co-exposure to PVC-MPs and Cd promote apoptosis in duck liver. Ducks were co-exposed to drinking PVC-MPs and dietary Cd for 60 d. (A) TUNEL staining was used to detect apoptosis in liver tissue of each group. Immunohistochemistry was used to detect expression level of Caspase-3 and Caspase-9 in liver tissue of each group. (Scale bars = 100 µm and 20 µm). (B-E) Levels of Bax and Bcl-2 were determined using western blotting. (B) Representative western blot image. (C-E) Quantitative analysis of B. The values are expressed as the mean ± SD (n = 4). Note: “*” represents a statistically significant difference between the Cd group and the control group (*P < 0.05, **P < 0.01 or ***P < 0.001), “^#^” represents a statistically significant difference between the Cd group and Cd + MPs group (^#^*P* < 0.05, ^##^*P* < 0.01, or ^###^*P* < 0.001). The meaning of the “*” and “^#^” remains the same throughout the text. “^#^” remains the same throughout the text.

TUNEL staining, immunohistochemistry, and Western Boltting were used to detect the effects of co-exposure to PVC-MP and Cd on apoptosis in liver tissues. The results of TUNEL staining showed that the proportion of apoptosis-positive cells was increased in the Cd group compared to the control group. The proportion of apoptosis-positive cells was significantly increased in the co-exposure to PVC-MP and Cd group compared to the Cd group. Immunohistochemical analysis showed increased immunosignaling for cysteine aspartate protease-3 (**Casepase-3**) and cysteine aspartate protease-9 (**Casepase-9**) in Cd-treated liver tissues compared to the control group. However, the immunosignaling of Casepase-3 and Casepase-9 was significantly increased in the co-exposure to PVC-MPs and Cd group compared to the Cd group (Figure 7A). The expression of apoptotic proteins in liver tissues of each group was further detected by Western Bolting. The results showed that Cd significantly increased the protein expression levels of Bax and decreased the protein expression levels of Bcl-2 compared to the control group. However, the changes in apoptosis-related protein markers were more severe in the co-exposure to PVC-MP and Cd group compared to the Cd group (Figures 7B-E).

## DISCUSSION

The pervasive usage of plastics and the concomitant increase in human exposure have led to a growing awareness of the adverse effects of plastics. Cd is recognized as a major pollutant in the natural environment. Long-term exposure to high concentrations Cd exerts adverse effects on the metabolism of animals. The liver is the largest detoxification and metabolic organ in the human body and is involved in numerous vital functions. The toxic effects of MPS on the liver are receiving increasing attention from researchers. Given that MP-related hepatotoxicity has been studied in a variety of species, including humans, mice, rats, oysters, and zebrafish, among others( Lu et al., 2016; Yang et al., 2022; Liu et al., 2023c; Missawi et al., 2023; Zarus et al., 2023), it is evident that the liver is a key organ affected by MP. However, regrettably, there are few studies investigating the mechanism of MP and Cd-induced hepatotoxicity in birds.

In recent years, MP have gained widespread attention as a novel environmental pollutant. It has been reported that MP residues were found in the yolk and liver of sea turtles at late embryonic stages and were PP and PVC, which were generally smaller than 5 μm in size (Chemello et al., 2023). Another study demonstrated a twofold increase in liver Cd accumulation when zebrafish were co-exposed to virgin or aged PE-MP and Cd (50 μg/L) for 21 d (Al Marshoudi et al., 2023). In our current study, co-exposure of PVC-MP with Cd resulted in a significant decrease in duck liver weight and impaired liver function. This report presents the first evidence that co-exposure of PVC-MP with Cd results in a significant increase in the accumulation of both PVC-MP and Cd in duck liver. This may be a contributing factor to the enhanced toxicity observed in co-exposure. Furthermore, we observed that PVC-MP mixed with Cd exhibited significant folds on the surface of MP at low concentrations, and at high concentrations, they split into MP with small particle sizes. A previous study demonstrated that MP with smaller particle sizes exhibited higher Cd adsorption capacity, and the adsorbed Cd was easily dissociated from the MP (Wang et al., 2019). This may explain why both PVC-MP and Cd were significantly elevated in the co-exposure model.

The majority of currently published literature indicates that the mechanism of toxicity of MP and Cd is typically associated with oxidative stress. Oxidative stress is initiated by an imbalance in the antioxidant system and the overproduction of ROS. Exposure of zebrafish embryos to 500 μg/L MPs with 5 μg/L Cd for 30 d resulted in an increase in ROS levels, an increase in MDA content, and a decrease in the activities of T-AOC, manganese superoxide dismutase (**Mn-SOD**), and CAT, indicating that co-exposure enhanced oxidative damage in zebrafish (Chen et al., 2022). The results of this study indicate that the co-exposure of PVC-MP and Cd groups resulted in a decrease in the antioxidant enzyme activities of GSH and SOD, an increase in the oxidase activity of CAT, and an elevation in the level of MDA compared to the control group. MDA is a significant byproduct of membrane lipid peroxidation. This damage may be attributed to the enhanced activity of CAT, which may be a response to the stimulation of excessive ROS within the cells (Lei et al., 2018). Cu, Mn, and Zn-superoxide dismutase are different isoforms of SOD. The reduced levels of Cu, Zn, and Mn in the liver of aged animals may have an effect on SOD and CAT activities (Popović et al., 2018). The results demonstrated a reduction in the elemental Mn content and revealed mitochondrial ultrastructural observations indicative of mitochondrial cristae breakage, vacuolization, and disruption of the bilayer membrane structure. These findings collectively indicate that co-exposure of PVC-MP with Cd results in significant oxidative damage in duck liver.

NAFLD is a significant contributor to chronic liver injury. However, the underlying mechanisms of its development remain poorly understood. Several lines of evidence indicate that the pathogenesis of NAFLD may be regulated by MP and NP that are absorbed or inhaled by humans (Auguet et al., 2022). Furthermore, it has been proposed that reducing the intake of MP, modulating the intestinal microbiota, or targeting the enterohepatic axis pathway may represent potential therapeutic strategies for NAFLD (Auguet et al., 2022). The results of this study indicate that co-exposure of PVC-MPs with Cd significantly increases glycogen content by inhibiting oxidative metabolism of glucose and activating the glycolytic pathway. A previous study demonstrated that PCK1 plays a pivotal role in the progression of NAFLD. Downregulation of PCK1 protein expression was observed in both NAFLD patients and animal models of mice fed a high-fat diet and deficiency of the glycoheterotrophic enzyme PCK1 may promote NAFLD in male mice via the PI3K/AKT/PDGF axis (Ye et al., 2023). Furthermore, our experiments indicated that PVC-MP and Cd co-exposure may facilitate duck liver glycogen synthesis through the PCK1-PI3K/AKT signaling pathway. So, these results indicated that MPs supplementation might activate PCK1/PI3K/AKT signaling pathway and enhance liver damage associated with Cd exposure. It has been previously reported in the literature that exposure to PP-MP resulted in significant alterations to lipid metabolic profiles, with notable increases in triglyceride, fatty acid, free fatty acid, and lysophosphatidylcholine content (Liu et al., 2023b). Our findings demonstrated that co-exposure of PVC-MP with Cd led to a pronounced increase in the number of liver tissue and cellular lipid droplets, accompanied by a significant elevation in TG and T-CHO content. Furthermore, NAFLD progression to later stages is characterized by the presence of liver fibers and hepatocellular carcinoma. Currently, it has been demonstrated that PVC-MP exposure is more likely to contribute to liver fibrosis and hepatocellular carcinoma incidence in MPs-susceptible workers (Zarus et al., 2023). Therefore, we examined liver fibrosis-related indicators and demonstrated that PVC-MPs and Cd exposure alone or in combination induced liver fibrosis, and that the combined exposure significantly enhanced the level of hepatic fibers compared with the exposure alone. In addition, chromatin consolidation, deepening of staining, and increase in the size of cell nuclei were observed due to previous tissue ultrastructure, and these features were consistent with apoptosis. Therefore, we examined apoptosis-related indicators and showed that apoptosis was induced by exposure to PVC-MP and Cd alone or in combination, and that the combined exposure significantly enhanced apoptosis levels compared with exposure alone. Currently, there are various modes of cell death, and it has been reported in the literature that MP induce apoptosis, necrosis, pyroptosis, and iron death in various organs and tissues of mice (Wu et al., 2024). Therefore, it is particularly important to further investigate the cell death modes of combined exposure to PVC-MP and Cd. Overall, our results open up new possibilities for exploring the mechanism of hepatotoxicity of PVC-MP co-exposed with Cd in female birds.

## CONCLUSION

The results of our study indicate that PVC-MP can accumulate in the liver of female breeding ducks. Furthermore, co-exposure significantly enhanced Cd bioaccumulation, which may exacerbate the combined toxicity of PVC-MP and Cd in female ducks. Co-exposure of PVC-MP with Cd led to subsequent changes in glycolipid metabolism, which were observed through Oxidative stress. Furthermore, co-exposure of PVC-MP with Cd may induce hepatic glycolipid accumulation through the PCK1-PI3K/AKT signaling axis, which may subsequently lead to hepatic fibrosis. These factors collectively resulted in hepatocyte apoptosis. Our findings contribute to the understanding of the combined toxicity of PVC-MP and Cd, which has significant implications for avian liver digestive health. However, the interaction among oxidative stress, fibrosis, and glycolipid metabolism disorder in MP and Cd-induced hepatotoxicity is complicated, and the results of animal experiments alone are not enough to explain the problem. The specific molecular mechanism and clinical significance of MP and Cd-induced hepatotoxicity need to be further verified by cell experiments.

## DISCLOSURES

The authors declare that they have no known competing financial interests or personal relationships that could have appeared to influence the work reported in this paper.
